# Mitotic transformation of TRAMM/TrappC12

**DOI:** 10.18632/oncotarget.4843

**Published:** 2015-07-11

**Authors:** Miroslav P. Milev, Michael Sacher

**Affiliations:** Concordia University, Department of Biology; McGill University, Department of Anatomy and Cell Biology, Montreal, Quebec, Canada

Intracellular vesicle transport is a mechanism required for the proper targeting and secretion of proteins and lipids. The process comprises sorting, budding, movement, tethering and fusion of the vesicle with the target membrane resulting in delivery of its contents [1].

Transport protein particle (TRAPP) has been demonstrated to contribute to endoplasmic reticulum (ER)-to–Golgi, intra-Golgi and late Golgi anterograde membrane transport as well as autophagy in both yeast and higher eukaryotes. In mammals two related complexes have been described called TRAPP II and TRAPP III [2]. While these complexes share a core of proteins, they each contain unique subunits, some of which are not found in yeast. Alterations of TRAPP subunits have been implicated in human diseases including spondyloepiphyseal dysplasia tarda, limb-girdle muscular dystrophy and a disorder including muscular, intellectual and kinetic phenotypes [3].

Our previous studies implicated the TRAPP subunits TrappC2, TrappC2L, TrappC8, TrappC11 and TrappC12 in an early stage of ER-to-Golgi transport and Golgi integrity [4,5].

Interestingly, several TRAPP complex components have other non-trafficking related functions, perhaps as a result of a TRAPP-independent mode of activity [3]. For example, the core component TrappC4 was implicated in tumorigenesis of colorectal cancer. The protein interacts with ERK2, modulates the nuclear localization of phospho-ERK2 and regulates proliferation and apoptosis in colorectal cancer cells. TrappC4 depletion induces cell cycle arrest in the G0/G1 phase and downregulates the expression of cyclin B1. TrappC9 has been implicated in NF-κB signaling via interactions with both NIK and IKK kinases. In addition, depletion of TrappC9 prevented nerve growth factor-induced neurite outgrowth. Thus, it may not be unexpected if other TRAPP subunits function in non-membrane trafficking processes.

Mitosis involves a complex sequence of events that is tightly regulated in space and time and is essential for genome inheritance. It provides accurate chromosome segregation which depends on interactions between microtubules and kinetochores, large protein structures localized at the centromere. CENP-E is a microtubule-dependent plus-end-directed motor expressed during mitosis and essential for congression of initially misaligned chromosomes prior to metaphase. CENP-E binds to a subset of kinetochore proteins that mediate the spindle assembly checkpoint (SAC), a critical mechanism that ensures proper chromosome alignment before anaphase. Disruption of CENP-E function leads to an accumulation of mitotically arrested cells with a population of chromosomes unaligned and in proximity to either of the spindle poles by obstructing microtubule plus-end-directed motion of chromosomes to the metaphase plate and preventing kinetochores from forming stable attachments with microtubules.

Our recent work identified and explored a novel function for TrappC12 in mitosis [6]. We demonstrated that TrappC12 (renamed to TRAMM; trafficking of membranes and mitosis), is a novel mitotic regulator with an important role in kinetochore assembly and stability that controls the recruitment of CENP-E to the kinetochores.

Our study began with the observation that TRAMM depletion, but not of any other TRAPP subunit, resulted in a dramatic increase in the mitotic index. Detailed analysis of mitotically arrested cells revealed that there was a chromosome congression defect highly similar to the CENP-E depletion phenotype. Importantly, TRAMM depletion led to the activation of SAC. Previously, using confocal microscopy, we observed that TRAMM mainly localizes to structures resembling the Golgi [5]. However, traces of the protein were also visible in the nucleus. Sub-cellular fractionation of interphase cells revealed that small amounts of TRAMM fractionated with nuclear components. Furthermore, TRAMM associated with isolated metaphase chromosomes and was loosely localized to the kinetochore. The kinetochore localization, in addition to the chromosome congression defect, suggested the protein may affect kinetochore structure. Indeed, depletion of TRAMM altered the recruitment of several key components to the kinetochore with CENP-E being the most dramatically affected. A recruitment assay further demonstrated that TRAMM is required for CENP-E association to the kinetochore.

If TRAMM, but no other TRAPP subunit, is involved in mitosis, we speculated that the protein must be released from the complex during mitosis. TRAMM is part of the TRAPP complex during interphase but during mitosis it becomes phosphorylated. Consistent with our hypothesis, size exclusion chromatography analysis revealed that TRAMM was no longer associated with the TRAPP complex and fractionated at a smaller molecular size, perhaps as a result of its phosphorylation. Phosphorylation of TRAMM began as the cells entered mitosis and was complete at the onset of anaphase. This temporal phosphorylation correlated with the localization patterns of TRAMM and CENP-E; maximal co-localization was detected during mitotic phosphorylation of TRAMM while distinct localization of the two proteins was apparent from anaphase onwards. After anaphase, TRAMM re-localized to the Golgi complex, presumably to participate in membrane trafficking, while CENP-E localized to the midbody.

A subset of membrane trafficking proteins, now including TRAMM, have been reported to be involved in mitosis [7]. However, TRAMM appears to be unique in that it cycles between two large complexes to perform its functions. Future investigations will be necessary to depict the precise mechanism and role of TRAMM in CENP-E recruitment. In particular, the role of its TPR domain, not involved in CENP-E binding (unpublished observation), will be important to address since this is a well-described protein-protein interaction domain. Finally, it will be important to define how TRAMM is released from TRAPP and re-incorporated into the complex, as well as potential non-TRAPP roles for other TRAPP subunits during the cell cycle.
